# The Role of Stereotactic Radiosurgery in the Management of Foramen Magnum Meningiomas—A Multicenter Analysis and Review of the Literature

**DOI:** 10.3390/cancers14020341

**Published:** 2022-01-11

**Authors:** Felix Ehret, Markus Kufeld, Christoph Fürweger, Alfred Haidenberger, Susanne Fichte, Ralph Lehrke, Carolin Senger, David Kaul, Martin Bleif, Gerd Becker, Daniel Rueß, Maximilian Ruge, Christian Schichor, Jörg-Christian Tonn, Alexander Muacevic

**Affiliations:** 1 Berlin Institute of Health at Charité—Universitätsmedizin Berlin, 10117 Berlin, Germany; 2 Charité—Universitätsmedizin Berlin, Corporate Member of Freie Universität Berlin and Humboldt-Universität zu Berlin, Department of Radiation Oncology, 13353 Berlin, Germany; carolin.senger@charite.de (C.S.); david.kaul@charite.de (D.K.); 3 European Radiosurgery Center, 81377 Munich, Germany; markus.kufeld@erc-munich.com (M.K.); christoph.fuerweger@erc-munich.com (C.F.); alfred.haidenberger@erc-munich.com (A.H.); alexander.muacevic@erc-munich.com (A.M.); 4 Department of Stereotaxy and Functional Neurosurgery, Center for Neurosurgery, University Hospital Cologne, 50937 Cologne, Germany; daniel.ruess@uk-koeln.de (D.R.); maximilian.ruge@uk-koeln.de (M.R.); 5 CyberKnife Center Mitteldeutschland, 99089 Erfurt, Germany; susanne.fichte@ckcm.de; 6 German CyberKnife Center, 59494 Soest, Germany; rlehrke@barbaraklinik.de; 7 Charité—Universitätsmedizin Berlin, Corporate Member of Freie Universität Berlin and Humboldt-Universität zu Berlin, Charité CyberKnife Center, 13353 Berlin, Germany; 8 German Cancer Consortium (DKTK), Partner Site Berlin, German Cancer Research Center (DKFZ), 69120 Heidelberg, Germany; 9 Radiochirurgicum, 73035 Göppingen, Germany; bleif@radiochirurgicum.de (M.B.); gerd.becker@af-k.de (G.B.); 10 Department of Neurosurgery, Ludwig-Maximilians-University Munich, 81377 Munich, Germany; christian.schichor@med.uni-muenchen.de (C.S.); joerg.christian.tonn@med.uni-muenchen.de (J.-C.T.)

**Keywords:** radiosurgery, stereotactic radiosurgery, meningioma, foramen magnum, robotic radiosurgery, CyberKnife, neuro-oncology, literature review, review

## Abstract

**Simple Summary:**

Meningiomas represent the most common central nervous system (CNS) tumor. Despite their often benign nature, a tumor location in direct proximity to vital brain structures may lead to significant morbidity. This is the case for foramen magnum meningiomas (FMMs) as they grow at the skull base, next to the brain stem and foramen magnum. Surgical resection represents the mainstay of FMM treatments. In patients unsuitable for surgery, with tumor recurrences or tumor remnants after surgery, non-invasive treatment modalities may play a crucial role in patient management. Reports and studies on stereotactic radiosurgery (SRS) for the treatment of FMMs are scarce. This multicenter analysis reported the outcome data of 62 patients with FMMs. SRS achieved a high local tumor control and demonstrated a favorable safety profile. These results are in agreement with previous findings. SRS should be considered for selected FMM patients.

**Abstract:**

Background: Foramen magnum meningiomas (FMMs) represent a considerable neurosurgical challenge given their location and potential morbidity. Stereotactic radiosurgery (SRS) is an established non-invasive treatment modality for various benign and malignant brain tumors. However, reports on single-session or multisession SRS for the management and treatment of FMMs are exceedingly rare. We report the largest FMM SRS series to date and describe our multicenter treatment experience utilizing robotic radiosurgery. Methods: Patients who underwent SRS between 2005 and 2020 as a treatment for a FMM at six different centers were eligible for analysis. Results: Sixty-two patients met the inclusion criteria. The median follow-up was 28.9 months. The median prescription dose and isodose line were 14 Gy and 70%, respectively. Single-session SRS accounted for 81% of treatments. The remaining patients received three to five fractions, with doses ranging from 19.5 to 25 Gy. Ten (16%) patients were treated for a tumor recurrence after surgery, and thirteen (21%) underwent adjuvant treatment. The remaining 39 FMMs (63%) received SRS as their primary treatment. For patients with an upfront surgical resection, histopathological examination revealed 22 World Health Organization grade I tumors and one grade II FMM. The median tumor volume was 2.6 cubic centimeters. No local failures were observed throughout the available follow-up, including patients with a follow-up ≥ five years (16 patients), leading to an overall local control of 100%. Tumor volume significantly decreased after treatment, with a median volume reduction of 21% at the last available follow-up (*p* < 0.01). The one-, three-, and five-year progression-free survival were 100%, 96.6%, and 93.0%, respectively. Most patients showed stable (47%) or improved (21%) neurological deficits at the last follow-up. No high-grade adverse events were observed. Conclusions: SRS is an effective and safe treatment modality for FMMs. Despite the paucity of available data and previous reports, SRS should be considered for selected patients, especially those with subtotal tumor resections, recurrences, and patients not suitable for surgery.

## 1. Introduction

Meningiomas are the most frequent central nervous system (CNS) tumors [1]. They usually occur at the convexity, falx, and parasagittally [2]. Only around 3% of all meningiomas arise at or close to the foramen magnum [3]. While surgery is the mainstay of treatment for meningiomas, foramen magnum meningiomas (FMMs) represent a considerable neurosurgical challenge given the risk for incomplete resections, surrounding anatomical structures, and potential for treatment-associated morbidity [4,5,6]. This is especially true for anterior and anterolateral FMMs, which comprise approximately 85% of such tumors. Depending on the surgeon’s experience and chosen surgical approach, the rates of permanent morbidity can be as high as 15 to 30%, with a peri- and postoperative mortality ranging between 0% and 25% [4,5]. The overall mortality in the past decades is around 6% [4]. If either complete surgical resection is not deemed feasible or the patient has a poor performance status, declines surgery, or in the case of a recurrence, stereotactic radiosurgery (SRS) may be a potential treatment alternative [6,7]. Given the rarity of FMMs and the sparse data available on the effectiveness and safety of this treatment modality, its role in the management of FMMs remains unclear [5,7]. The available literature is mostly based on case reports and smaller case series, most of them reporting treatment outcomes for GammaKnife (GK)-based SRS. Herein, we aim to provide more evidence on the use of SRS with robotic radiosurgery (RRS), utilizing a national multicenter analysis. RRS applies non-isocentric and non-coplanar treatment beams with high precision without the necessity of a stereotactic frame [8]. We compare our RRS results to the existing data and review the respective literature.

## 2. Materials and Methods

Patients who were treated with SRS for a FMM between 2005 and 2020 were eligible for analysis. Six institutions participated in this retrospective multicenter analysis. Diagnosis of an FMM was either confirmed by (1) histopathological examination after surgery or biopsy or (2) radiographic appearance with respective clinical behavior, or both, and validated by an interdisciplinary neuro-oncological tumor board involving neurosurgeons, neuroradiologists, neuropathologists, and radiation oncologists. Only histopathologically confirmed or radiographically suggested Word Health Organization (WHO) grade I or II meningiomas were included. Malignant meningiomas (WHO grade III) were excluded.

Anatomical classification of FMMs in regard to the compartment of development and dural insertion was performed according to the work of Bruneau and George [4]. Previous treatments, neurological deficits, imaging data, histology, and performance status were recorded prior to SRS treatment. Only patients with at least one combined radiographic and clinical follow-up were included. All patients underwent SRS with up to five fractions using a CyberKnife^®^ robotic radiosurgery system (Accuray Inc., Sunnyvale, CA, USA). For treatment delivery, 1 mm computed tomography (CT) and 1 mm contrast-enhanced MRI scans were acquired and subsequently merged for inverse treatment planning with a dedicated planning software (MultiPlan^®^, Precision^®^, Accuray Inc., Sunnyvale, CA, USA). For target definition, the visible tumor on contrast-enhanced imaging was contoured as the target volume, with no additional margin for the planning target volume (PTV). The treatment dose and number of fractions were selected according to each institution’s standard and at the discretion of the treating physicians. Suggested dose constraints of the American Association of Physicists in Medicine Task Group 101 (AAPM TG 101) were applied if technically and medically appropriate and subject to modifications for individual cases [9]. The maximum dose for the pons, medulla oblongata, and spinal cord were summarized for comparability and to account for the varying tumor expansion.

Local tumor response, neurological deficits, and adverse events (AE) were evaluated clinically and by imaging follow-up according to each center’s preferences and depending on the patient’s clinical status. Local control (LC) was defined as the absence of tumor growth of more than 10% of the pretreatment volume of the irradiated FMM on follow-up imaging (CT/MRI). Tumor response was defined as a > 10% volume decrease at the last available follow-up. Tumor progression was defined as a > 10% volume increase during the available follow-up imaging.

Progression-free survival (PFS) was calculated from the time of RRS until death, progression, or the last follow-up using the Kaplan–Meier estimate. Log-rank tests were used to compare time-to-event variables such as time of LC and PFS between single-session and multisession treatments. Data were tested for normal distribution using the Shapiro–Wilk test and graphical appearance, including skewness and kurtosis. Respective analyses were performed using paired and unpaired student’s t-test and Wilcoxon rank-sum and signed-rank tests. All *p*-values were two-sided, with an α-level of 0.05. Statistical analyses were performed with STATA MP 16.0 (StataCorp, College Station, TX, USA). 

A PubMed-based literature search was conducted to identify published reports and studies on the use of SRS for FMMs. Only studies with full-body texts in English which reported the radiosurgical treatment with up to five fractions until 31 July 2021 were reviewed. If studies also reported on the treatment of other tumor entities, data for FMMs were extracted if possible.

## 3. Results

### 3.1. Patient and Treatment Characteristics

Sixty-two patients with 62 FMMs were included in this analysis. Forty-four patients (71%) were female, and the median age at treatment delivery was 58.5 years. The median Karnofsky Performance Status (KPS) was 100%. One patient was suffering from a neurofibromatosis type II. Twenty-three patients (37%) underwent at least one surgical resection before SRS. None of the included patients received a radiotherapy before SRS. Ten (16%) were treated for a tumor recurrence, and thirteen patients (21%) underwent adjuvant treatment after incomplete tumor resection. The remaining 39 patients (63%) underwent SRS as their primary treatment.

The majority of FMMs were located in the intradural compartment (68%). The lateral location was the most frequent one (34%). For the 23 patients who underwent surgery, histopathological examination showed 22 World Health Organization (WHO) grade I and one grade II FMM.

Before SRS, the most common symptoms included headaches (16%), vertigo (15%), hypoesthesia (13%), and motoric deficits (13%). One patient experienced a cerebrospinal fluid fistula after surgical resection. Eighteen patients (29%) did not show neurological deficits before SRS. The patient characteristics are summarized in Table 1. The pre- and posttreatment symptoms are summarized in Table 2.

The median prescription dose and isodose line were 14 Gy and 70%, respectively. Fifty out of 62 tumors (81%) were treated with one fraction. The remaining received three to five fractions, with doses ranging from 19.5 to 25 Gy. The median tumor volumes of primarily and secondarily treated tumors were 2.2 cubic centimeters (cc) and 3.9 cc, respectively. Tumor volumes for single-session treatments were significantly smaller compared to multisession SRS (median sizes: 2.1 versus 5.3 cc, *p* < 0.01). The median maximum dose to the brain stem was 13.5 Gy. The treatment characteristics are summarized in Table 3.

### 3.2. Treatment Outcomes

**Figure 1:**
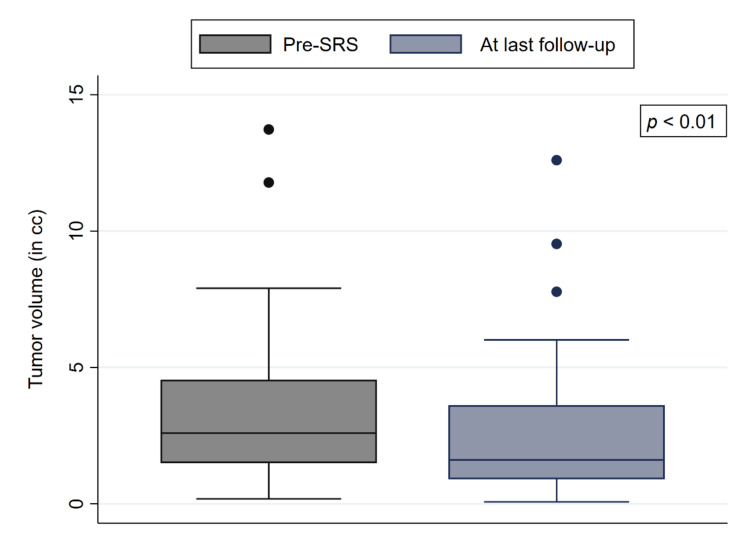
Box plots of the pretreatment and posttreatment tumor volumes.

**Figure 2:**
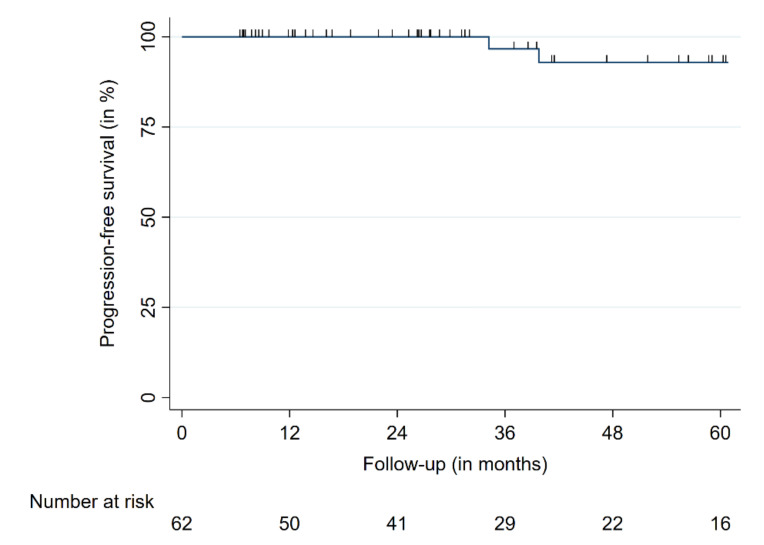
Progression-free survival.

At a median follow-up of 28.9 months, all 62 tumors were controlled, including patients with a follow-up ≥ five years (16 patients), leading to an overall LC of 100%. Forty-three tumors (69%) showed a significant volume decrease of 10% or more (*p* < 0.01) (Figure 1). Nineteen tumors (31%) did not show a change of tumor volume ±10%. Three tumors (5%) slightly increased in size (+1, +2, and +3% of the respective tumor volumes). We observed four deaths in our patient cohort, none of them related to the FMM or SRS (two deaths from cardiovascular disease, one due to respiratory failure secondary to end stage chronic obstructive pulmonary disease, and one death was caused by a metastasized malignant melanoma). The one-, three-, and five-year PFS were 100%, 96.6%, and 93.0%, respectively (Figure 2).

**Figure 3:**
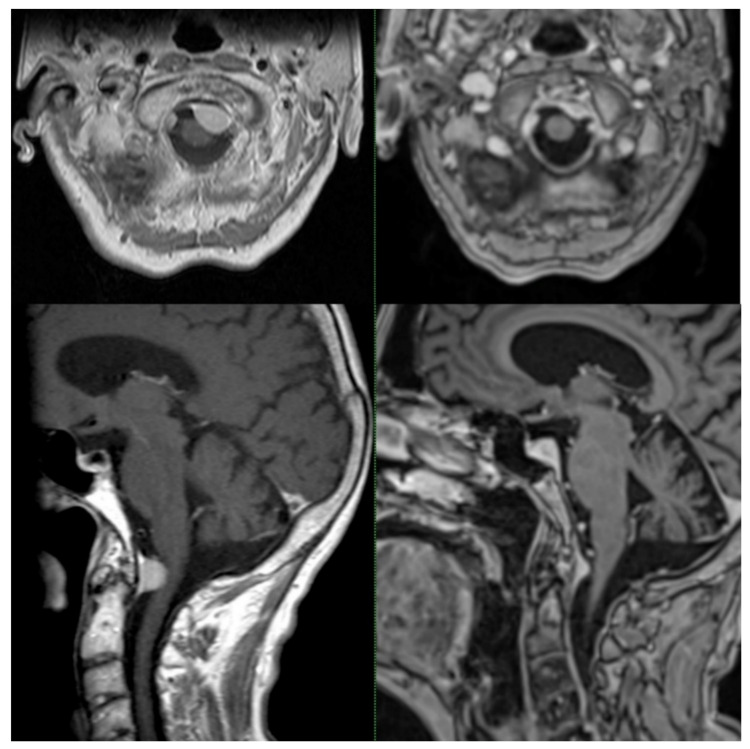
Axial and sagittal pretreatment (left top and bottom) and posttreatment contrast-enhanced MRI (right top and bottom). Incidental finding of a FMM in a 79-year-old man without any neurological findings. To avoid future tumor-associated complications and with respect to the age and comorbidities of the patient, SRS of the FMM was conducted in one session with 13.5 Gy prescribed to the 70% isodose line. After a follow-up of 67 months, the tumor volume decreased from 2.1 to 0.6 cc. No new neurological deficits developed after treatment delivery.

Regarding the clinical outcome, thirteen patients (21%) with pretreatment deficits reported an improvement at the last follow-up. Of these, seven patients (11%) recovered entirely. None of the eighteen patients (29%) without pre-SRS deficits developed neurological symptoms after treatment. Most patients (47%) showed no significant change concerning their pretreatment symptoms. Two patients (3%) had worsening of preexisting symptoms or developed new neurological deficits, including a new onset of trapezius muscle weakness and new absence seizures, both occurring after SRS. No radiation necrosis, bleeding, or treatment-induced malignancy were observed. No differences regarding tumor volume reduction or toxicity between single-session and multisession treatments were noted. A representative case is shown in Figure 3.

### 3.3. Literature Review

Twelve studies were identified. Four studies (33%) did not exclusively report on the treatment of FMMs. Only two other studies with a total of eight FMMs described outcomes after RRS, with most reports (83%) utilizing GK-based SRS [10,11]. Overall, approximately 100 unique FMM treatments with SRS have been reported to date. The exact number cannot be determined given multiple reports from same institutions and small, unidentifiable patient subgroups in larger SRS meningioma studies.

Median applied doses were between 12 and 15.2 Gy for single-session treatments, with multisession treatments being rarely reported. LC rates ranged between 80% and 100% in the reviewed studies. However, most series showed a favorable LC of more than 90% throughout an intermediate follow-up. The majority of patients showed either a symptom improvement or stable neurological deficits. The treatment was well tolerated, and AE were scarce. Notably, the reported patient data were heterogeneous concerning pretreatments, neurological deficits, and the other tumor types included. The studies and outcomes are summarized in Table 4.

## 4. Discussion

FMMs represent a rare but considerable neuro-oncological challenge given their location with direct involvement of lower cranial nerves, the intradural segment of the vertebral arteries [6,7]. Surgery remains the mainstay of treatment for meningiomas including FMMs but given the considerable risk for treatment-associated morbidity and mortality, non-invasive treatment may embody a viable alternative [4,6,21]. Yet, radiotherapy and SRS for this tumor entity are infrequently applied, and only sparse data are available to sufficiently define their role in the management. Herein, we report our experience of more than 15 years of treating FMMs with SRS utilizing RRS.

We observed a favorable local control (LC), with none of the FMMs showing a tumor progression after irradiation throughout an intermediate follow-up duration. This is in agreement with the previously published literature and larger case series [7,12,14,17]. The multicenter analysis of Mehta et al., representing the experience of the International Gamma Knife Research Foundation, was the largest SRS study to date [7]. Their results were also favorable, with a LC of 93% after a median radiographic follow-up of 36 months [7]. Tumor growth after irradiation is rare for WHO grade I tumors [20,22]. However, a limited duration of follow-up may mask late tumor progression or recurrences. Moreover, with recent advances in the field of DNA methylation-based tumor classifications, risk stratification is limited to histopathological and radiographic features [23,24]. Still, SRS and fractionated radiotherapy have shown considerable LC rates for meningiomas regardless of their location, and thus they represent a regular part of the multimodal treatment of this tumor entity, even as the primary treatment modality [21,25]. However, FMMs are close to vital structures like the brain stem, spinal cord, and medulla oblongata. This may explain the hesitation of radiation oncologists to treat these lesions with SRS or fractionated radiotherapy, given the anticipated risk for severe radiation injuries [26,27]. However, data suggest that SRS in this area is not only feasible, but relatively well tolerated by patients if dose constraints are respected [26,27].

In our analysis, we mostly followed the recommendations of the AAPM TG 101, with a maximum point dose of 15/23/31 Gy for the brain stem and 14/22/30 Gy for the spinal cord and medulla for single-session treatments/three fractions/five fractions [9]. Throughout our median follow-up of 29.8 months and a median max dose of 13.5 Gy to these organs at risk, we observed no radiation injury or significant toxicity, even for patients with an extensive follow-up of more than five years. This is also in agreement with the respective literature and studies reviewed herein (Table 4). Treatment of FMMs with SRS rarely caused AE.

Herein, we also report several cases treated with multisession SRS. There was no difference to single-session treatments regarding safety or treatment outcomes. Tumors treated with more than one fraction had larger volumes (median sizes: 5.3 versus 2.1 cc). Notably, the prescribed dose and number of fractions were at the discretion of the respective physicians and not standardized as this work is of retrospective nature. Fractionation may play an important role in previously irradiated tumors and those causing intolerable max doses to organs at risk during single-fraction treatment planning due to the total tumor volume [28]. This reasoning can be explained by the biologically effective dose and respective fractionation effect for benign tumors [29]. Such tumors are comparable with late-responding tissue and may profit from fractionated, high dose irradiations [29]. However, it remains unclear to which extent multisession SRS may improve treatment tolerability, especially concerning the organs at risk. Finally, we cannot draw firm conclusions on this matter given the small sample size and obtained results. Including this series, approximately 160 unique cases have been reported in the literature, highlighting the paucity of non-invasive treatment reports. Dedicated reports of conventionally fractionated radiotherapy are even scarcer [30]. Still, the results of this analysis, which include nearly 40% of all reported FMM SRS treatments, and previous case series are encouraging.

Achieving a tumor volume reduction or cessation of tumor growth are crucial long-term objectives in the treatment of this tumor type [31]. Patients suffering from FMMs should be carefully counseled, and the treatment must be tailored to the individual preferences of the patients in accordance with the performance status, neurological deficits, and potential risks. Despite the limited data available, we consider SRS a reasonable primary treatment alternative or addition to surgery in selected patients. More precisely, small- to medium-sized, incompletely resected, or recurring tumors are reasonable candidates for SRS. Besides, SRS may also be applied for patients who decline surgery. In addition, anteriorly and anterior-laterally located FMMs, which comprise the majority of tumors in our series, may lead to considerable morbidity and mortality during and after surgery [7,32]. Thus, the tumor location is another vital factor that must be addressed when choosing the treatment modality.

Limitations of this report include its retrospective nature, the associated sampling bias, follow-up duration, and limited sample size. Moreover, treatments were not completely standardized given the rarity of such tumors and individual preferences of each participating center. In addition, not all lesions were histopathologically confirmed despite striking radiographic and clinical evidence. Finally, tumor and risk stratification were limited to the WHO grading without further genetic or methylomic analysis.

## 5. Conclusions

SRS is an effective and safe treatment modality for FMMs. Despite the paucity of available data and previous reports, SRS may be considered for selected patients, especially those with subtotal tumor resections, recurrences, and patients not suitable for surgery.

## Figures and Tables

**Table 1:** Patient characteristics.

Number of patients	62				
Gender (male/female)	18		44		
%	29		71		
	**Median**	**Mean (** **±** **SD)**	**IQR**	**Range**	
Age (years)	58.5	60.1 (±14.5)	49.0–72.6	33.7–89.6	
Pretreatment Karnofsky Performance Status (%)	100	93.0 (10.3)	90–100	50–100	
Follow-up (months)	28.9	39.0 (±31.6)	12.3–60	5.9–132.2	
RRS indication	Primary treatment	Recurrence		Adjuvant treatment	
Number of patients	39	10		13	
%	63	16		21	
Tumor location	Anterior	Lateral	Posterior	PL	AL
Number of patients	14	21	4	9	14
%	23	34	6	14	23
Tumor compartment	Intradural	Intra-extradural		Extradural	
Number of patients	42	16		4	
%	68	26		6	
WHO tumor grading *	I		II		
Number of patients	22		1		
%	96		4		

SD = standard deviation, IQR = interquartile range, RRS = robotic radiosurgery, cc = cubic centimeters, Gy = Gray, WHO = World Health Organization, PL = postero-lateral, AL = antero-lateral. * = confirmed by histopathology.

**Table 2:** Symptoms before SRS (left columns) and at the last available follow-up (right columns).

Symptom/Deficit	Number of Patients	%	Number of Patients	%
Headaches	10	16	10	16
Vertigo	9	15	9	15
Hypoesthesia	8	13	7	11
Muscle weakness/paralysis extremities	8	13	8	13
XII neuropathy	5	8	2	3
Dysphagia	5	8	2	3
Neck pain	3	5	1	2
Hearing loss	3	5	2	3
Ataxia	3	5	2	3
Partial hearing loss	3	5	2	3
Dysesthesia	3	5	0	0
Seizures	2	3	3	5
XI neuropathy	1	2	0	0
VII neuropathy	1	2	1	2
CSF fistula	1	2	1	2
Tinnitus	1	2	0	0
Dysarthria	1	2	0	0

**Table 3:** Treatment characteristics.

Treatment Variable	Median	Mean (±SD)	IQR	Range
Tumor volume (cc)	2.6	3.3 (±2.6)	1.4–4.5	0.2–13.7
Prescription dose all treatments (Gy)	14	15.4 (±3.6)	13.5–15	12–25
Prescription dose single-session treatments (Gy)	14	13.8 (±1.0)	13–14.5	12–17
Prescription dose multisession treatments (Gy)	21	22.0 (±2.6)	19.5–25	19.5–26
Prescription isodose line (%)	70	70.6 (±4.3)	70–70	61–80
Number of fractions	1	1.5 (±1.2)	1–1	1–5
Max tumor dose (Gy)	20.0	21.8 (±5.4)	18.6–23.0	15.0–37.8
Mean tumor dose (Gy)	17.0	18.4 (±4.3)	15.8–18.7	13.4–31.5
Min tumor dose (Gy)	12.7	13.4 (±3.0)	11.8–13.9	8.9–21.1
Max dose medulla oblongata/brainstem (Gy)	13.5	14.0 (±4.2)	11.6–15.1	6.7–26.5
Conformity index	1.24	1.27 (±0.17)	1.17–1.36	1.05–1.94
Homogeneity index	1.43	1.42 (±0.09)	1.41–1.43	1.25–1.82
Coverage (%)	98.7	97.8 (±2.3)	97.1–99.2	87.4–100

SD = standard deviation, IQR = interquartile range, cc = cubic centimeters, Gy = Gray.

**Table 4:** Literature review on the treatment of FMMs with SRS.

Author	Year	Number of Patients	Number of Primarily Treated FMMs	Treatment Modality	Median Follow-Up in Months	Median Tumor Volume in cc	Dose/Fractions	Clinical Outcome	Radiographic Outcome
Mehta et al. [7] *	2017	57	37	GK	Radiographic: 36, clinical: 53	2.9	Median margin dose: 12.5 Gy, one fraction	Improvement: 52%	LC: 93%
Sheehan et al. [12] *, **	2015	18	NR	GK	60.1 (all patients)	6.5 (all tumors)	Median margin dose: 13.6 Gy, one fraction	Improvement or stable: 91% (all patients)	LC: 91% (all tumors)
Malone et al. [10]	2020	1	1	CK	97	14.2	30 Gy, five fractions	Improvement	LC: 100%
Cheshier et al. [11] ***	2007	7	NR	CK	Mean radiographic: 15.4, mean clinical: 8.9	1.5	18 Gy, two fractions	Improvement: 29%, stable: 46%, worse: 25% (for 24 patients)	LC: 83% for 23 patients (all tumors)
Muthukumar et al. [13]	1999	5	3	GK	36	10.5	Median margin dose: 14 Gy, one fraction	Stable: 100%	LC: 100%
Akyoldaş et al. [14]	2020	37	25	GK	Median radiographic: 84, median clinical: 80	3.3	Median margin dose: 12 Gy, one fraction	Improvement: 73%, Stable: 27%	LC: 97.3%
Mohammed et al. [15] *	2020	1	1	GK	61	5.8	13.5 Gy in one fraction	Stable	LC: 100%
Starke et al. [16] *	2010	5	2	GK	72	6.8	Median margin dose: 12 Gy, one fraction	Improvement: 60%, stable: 20%, worse: 20%	LC: 80%
Zenonos et al. [17] *	2012	21	15	GK	47	4.1	Median margin dose: 13 Gy, one fraction	Improvement: 48%, stable: 52%	LC: 100%
Nicolato et al. [18] ^†^	2001	1	NR	GK	28.7	Mean: 5.9	Mean margin dose: 15.2 Gy, one fraction	Improvement or stable: 100% (all patients)	LC: 95% (all tumors)
Lin et al. [19]	1998	1	0	GK	NR	NR	NR	Stable	NR
Kondziolka et al. [20] *, ^‡^	2008	22	NR	GK	48 (all patients)	Mean: 7.4 (all tumors)	Mean margin dose: 14 Gy, one fraction (all patients)	Improvement or stable: 91%/93% for WHO grade I/primary treatments (all patients)	LC: 93%/97% at the median follow-up for WHO grade I/primary treatments (all tumors)
This series	2021	62	39	CK	28	2.6	14 Gy, one fraction	Improvement: 21%, Stable: 47%, Worse: 3% No symptoms before and after SRS: 29%	LC: 100%

*: Overlapping data from same institutions, **: Study included a total of 675 posterior fossa meningiomas, ***: Study included 18 other benign and 10 malignant tumors, ^†^: Study included a total of 57 patients with 62 posterior fossa meningiomas, ^‡^: Study included a total of 972 patients with 1045 intracranial meningiomas. NR = Not reported, CK = CyberKnife, GK = GammaKnife.

## Data Availability

The data that support the findings of this study are available from the corresponding author, F.E., upon reasonable request.
